# Deletion of 8p is an independent prognostic parameter in prostate cancer

**DOI:** 10.18632/oncotarget.13425

**Published:** 2016-11-17

**Authors:** Martina Kluth, Nina Nadine Amschler, Rami Galal, Christina Möller-Koop, Phillipp Barrow, Maria Christina Tsourlakis, Frank Jacobsen, Andrea Hinsch, Corinna Wittmer, Stefan Steurer, Till Krech, Franziska Büscheck, Till Sebastian Clauditz, Burkhard Beyer, Waldemar Wilczak, Markus Graefen, Hartwig Huland, Sarah Minner, Thorsten Schlomm, Guido Sauter, Ronald Simon

**Affiliations:** ^1^ Institute of Pathology, Prostate Cancer Center at University Medical Center Hamburg-Eppendorf, Germany; ^2^ Martini-Clinic, Prostate Cancer Center at University Medical Center Hamburg-Eppendorf, Germany; ^3^ Department of Urology, Section for Prostate Cancer Research, University Medical Center Hamburg-Eppendorf, Germany

**Keywords:** NKX3.1, TMPRSS2:ERG, PTEN, prostate cancer, tissue microarray

## Abstract

Deletion of chromosome 8p is the second most frequent genomic alteration in prostate cancer. To better understand its clinical significance, 8p deletion was analyzed by fluorescence *in-situ* hybridization on a prostate cancer tissue microarray. 8p deletion was found in 2,581 of 7,017 cancers (36.8%), and was linked to unfavorable tumor phenotype. 8p deletion increased from 29.5% in 4,456 pT2 and 47.8% in 1,598 pT3a to 53.0% in 931 pT3b-pT4 cancers (*P* < 0,0001). Deletions of 8p were detected in 25.5% of 1,653 Gleason ≤ 3 + 3, 36.6% of 3,880 Gleason 3 + 4, 50.2% of 1,090 Gleason 4 + 3, and 51.1% of 354 Gleason ≥ 4 + 4 tumors (*P* < 0,0001). 8p deletions were strongly linked to biochemical recurrence (*P* < 0.0001) independently from established pre- and postoperative prognostic factors (*P* = 0.0100). However, analysis of morphologically defined subgroups revealed, that 8p deletion lacked prognostic significance in subgroups with very good (Gleason ≤ 3 + 3, 3 + 4 with ≤ 5% Gleason 4) or very poor prognosis (pT3b, Gleason ≥ 8, pN1). 8p deletions were markedly more frequent in cancers with (53.5%) than without *PTEN* deletions (36.4%; *P* < 0,0001) and were slightly more frequent in ERG-positive (40.9%) than in ERG-negative cancers (34.7%, *P* < 0.0001) due to the association with the ERG-associated *PTEN* deletion. Cancers with 8p/*PTEN* co-deletions had a strikingly worse prognosis than cancers with deletion of *PTEN* or 8p alone (*P* ≤ 0.0003). In summary, 8p deletion is an independent prognostic parameter in prostate cancer that may act synergistically with *PTEN* deletions. Even statistically independent prognostic biomarkers like 8p may have limited clinical impact in morphologically well defined high or low risk cancers.

## INTRODUCTION

Prostate cancer is the most prevalent cancer in men in Western societies [1]. Although the majority of prostate cancers behave in an indolent manner, a small subset is highly aggressive and requires extensive treatment [2, 3]. Established preoperative prognostic parameters are limited to Gleason grade, tumor extent on biopsies, prostate-specific antigen (PSA), and clinical stage. These data are statistically powerful, but often not sufficient to optimize individual treatment decisions. It is thus hoped that a better understanding of disease biology will eventually lead to the identification of clinically applicable molecular markers that enable a more reliable prediction of prostate cancer aggressiveness.

Deletions of chromosome 8p are of high interest in prostate cancer. After *TMPRSS2*:*ERG* fusion, 8p deletions constitute the second most frequent genomic alteration in this tumor type, occurring in about 30% of cancers [4–6]. A specific target gene of the 8p deletion has never been identified. It appears likely, that the tumorigenic effect of 8p deletions occurs through a combined downregulation of multiple relevant genes on 8p. A variety of studies have earlier analyzed 8p deletions in prostate cancer and reported associations with advanced tumor stage and Gleason grade [5–12], metastatic growth [8, 12], early biochemical recurrence [10, 13], and reduced overall survival [14]. Most of these studies were done on patient cohorts ranging from 27 to 195. In an own study on 2,097 cancers we had only found a marginal association with clinical outcome [10].

Because of the still not clarified role of this highly frequent alteration we decided to expand our earlier study in order to better assess the clinical significance of 8p deletion including subgroup analyses and comparisons with other relevant molecular features. For this purpose we took advantage of our current version of a prostate cancer tissue microarray (TMA) now containing more than 12,000 prostate cancers.

## RESULTS

### Architecture of 8p deletions in prostate cancer

**Figure 1:**
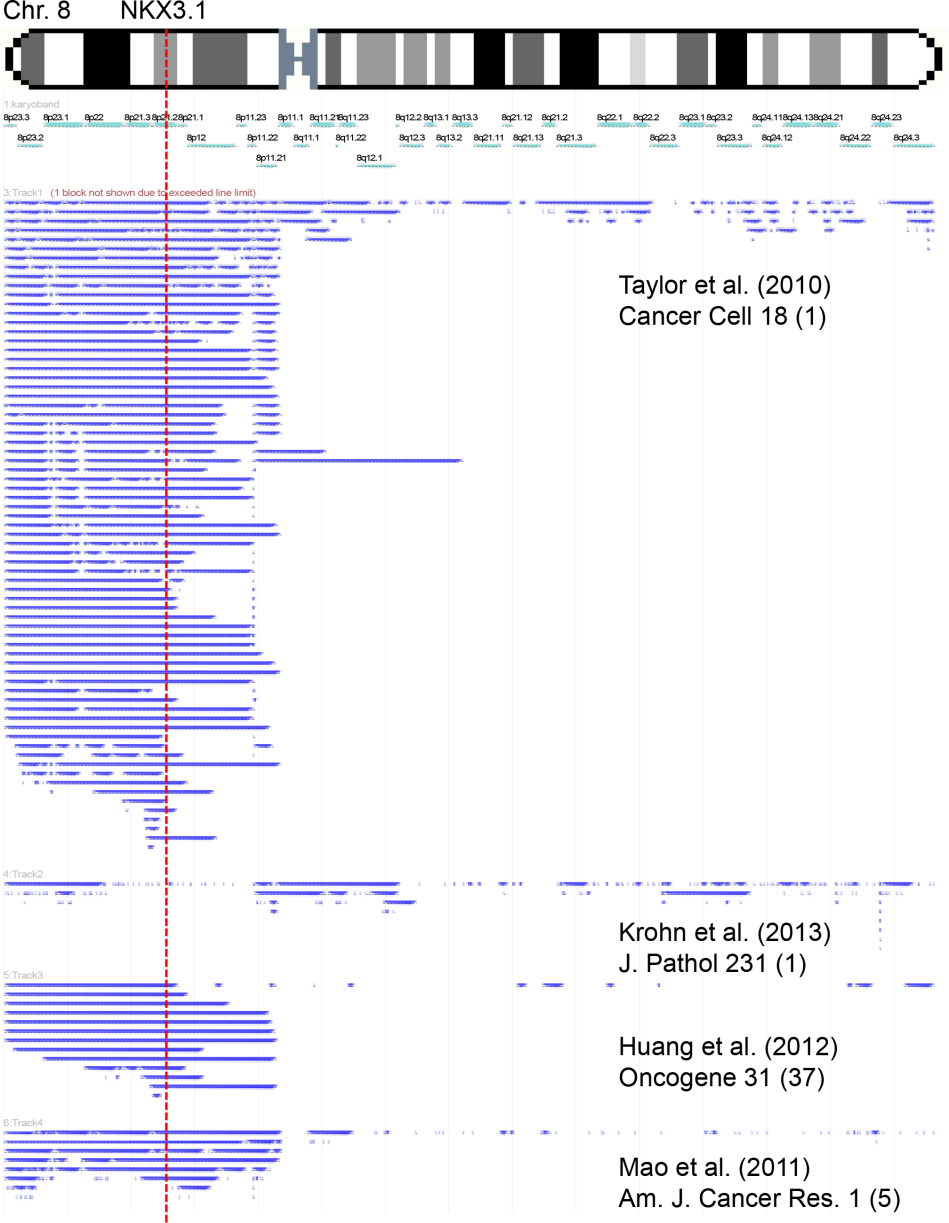
Size and extension of chromosome 8 deletions detected in published microarray-based copy number studies Each bar represents the deleted area in a single tumor.

Re-analysis of own [15] and published [4, 16, 17] microarray-based 8p copy number data from 442 prostate cancers using the FISH-Oracle browser [18, 19] shows that 8p deletions typically involve the entire short arm of chromosome 8 (Figure 1).

### Technical aspects

8p (*NKX3.1*) FISH analysis was successful in 7,017 of 12,427 (56.5%) arrayed cancers. Analysis was not informative in the remaining of 5,410 tumors because lack of tumor cells in the tissue spots, faint or absent FISH signals, or missing tissue spots on the TMA section.

### Prevalence and type of 8p deletions and association to prostate cancer phenotype

8p deletions were found in 36.8% (2,581/7,017) of all prostate cancers. All deletions were heterozygous. The relationship between 8p deletions and tumor phenotype and clinical parameters is summarized in Table 1. 8p deletions were significantly linked to advanced tumor stage, high Gleason grade, presence of lymph node metastasis, pre-surgical prostate specific antigen (PSA) level and positive surgical margin (*P* < 0.0001 each). All associations between 8p deletions and clinico-pathological variables held also true in the subsets of 3,569 ERG-negative (*P* < 0.0001) and 2,993 ERG-positive cancers (*P* ≤ 0.0005, Table 2).

**Table 1:** Associations between 8p deletion and prostate cancer phenotype in all cancers

		*n* analyzable	8p normal (%)	8p deletion (%)	*P*-value
**all cancers**		7017	4436 (63.2)	2581 (36.8)	
**tumor stage**	pT2	4456	3141 (70.5)	1315 (29.5)	< 0.0001
	pT3a	1598	835 (52.3)	763 (47.8)	
	pT3b-pT4	931	438 (47.1)	493 (53.0)	
**Gleason grade**	≤ 3 + 3	1653	1232 (74.5)	421 (25.5)	< 0.0001
	3 + 4	3880	2462 (63.5)	1418 (36.6)	
	4 + 3	1090	543 (49.8)	547 (50.2)	
	≥ 4 + 4	354	173 (48.9)	181 (51.1)	
**lymph node metastasis**	N0	3946	2386 (60.5)	1560 (39.5)	< 0.0001
	N+	398	178 (44.7)	220 (55.3)	
**PSA level (ng/μl)**	< 4	870	599 (68.9)	271 (31.2)	< 0.0001
	4–10	4138	2672 (64.6)	1466 (35.4)	
	10–20	1417	855 (60.3)	562 (39.7)	
	> 20	510	260 (51.0)	250 (49.0)	
**surgical margin**	negative	5559	3629 (65.3)	1930 (34.7)	< 0.0001
	positive	1334	726 (54.4)	608 (45.6)	

**Table 2:** Associations between 8p deletion and prostate cancer phenotype in the subgroup of ERG-positive and ERG-negative cancers

		ERG-positive cancers				ERG-negative cancers			
		*n* analyzable	8p normal (%)	8p deletion (%)	*P*-value	*n* analyzable	8p normal (%)	8p deletion (%)	*P*-value
all cancers		2993	1769 (59.1)	1224 (40.9)		3569	2332 (65.3)	1237 (34.7)	
tumor stage	pT2	1742	1158 (33.5)	584 (66.5)	< 0.0001	2400	1729 (72.0)	671 (28.0)	< 0.0001
	pT3a	815	417 (48.8)	398 (51.2)		701	373 (53.2)	328 (46.8)	
	pT3b-pT4	419	184 (56.1)	235 (43.9)		455	220 (48.4)	235 (51.7)	
Gleason grade	< 7	685	481 (70.2)	204 (29.8)	< 0.0001	814	619 (76.0)	195 (24.0)	< 0.0001
(WHO/ISUP 2016)	3+4	1743	1035 (59.4)	708 (40.6)		1940	1285 (66.2)	655 (33.8)	
	4+3	437	196 (44.9)	241 (55.1)		601	319 (53.1)	282 (46.9)	
	8	21	15 (71.4)	6 (28.6)		51	21 (41.2)	30 (58.8)	
	9–10	105	42 (40.0)	63 (60.0)		158	84 (53.2)	74 (46.8)	
lymph node metastasis	N0	1678	952 (56.7)	726 (43.3)	0.0002	2049	1287 (62.8)	762 (37.2)	< 0.0001
	N+	182	77 (42.3)	105 (57.7)		193	88 (45.6)	105 (54.4)	
PSA level (ng/μl)	< 4	409	261 (63.8)	148 (36.2)	0.0005	387	280 (72.4)	107 (27.7)	< 0.0001
	4–10	1800	1078 (59.9)	722 (40.1)		2071	1390 (67.1)	681 (32.9)	
	10–20	540	314 (58.2)	226 (41.9)		800	491 (61.4)	309 (38.6)	
	> 20	201	93 (46.3)	108 (53.7)		278	149 (53.6)	129 (46.4)	
surgical margin	negative	2320	1418 (61.1)	902 (38.9)	< 0.0001	2868	1933 (67.4)	935 (32.6)	< 0.0001
	positive	617	319 (51.7)	298 (48.3)		643	357 (55.5)	286 (44.5)	

### Association between 8p deletion, ERG fusion and PTEN deletion

**Figure 2:**
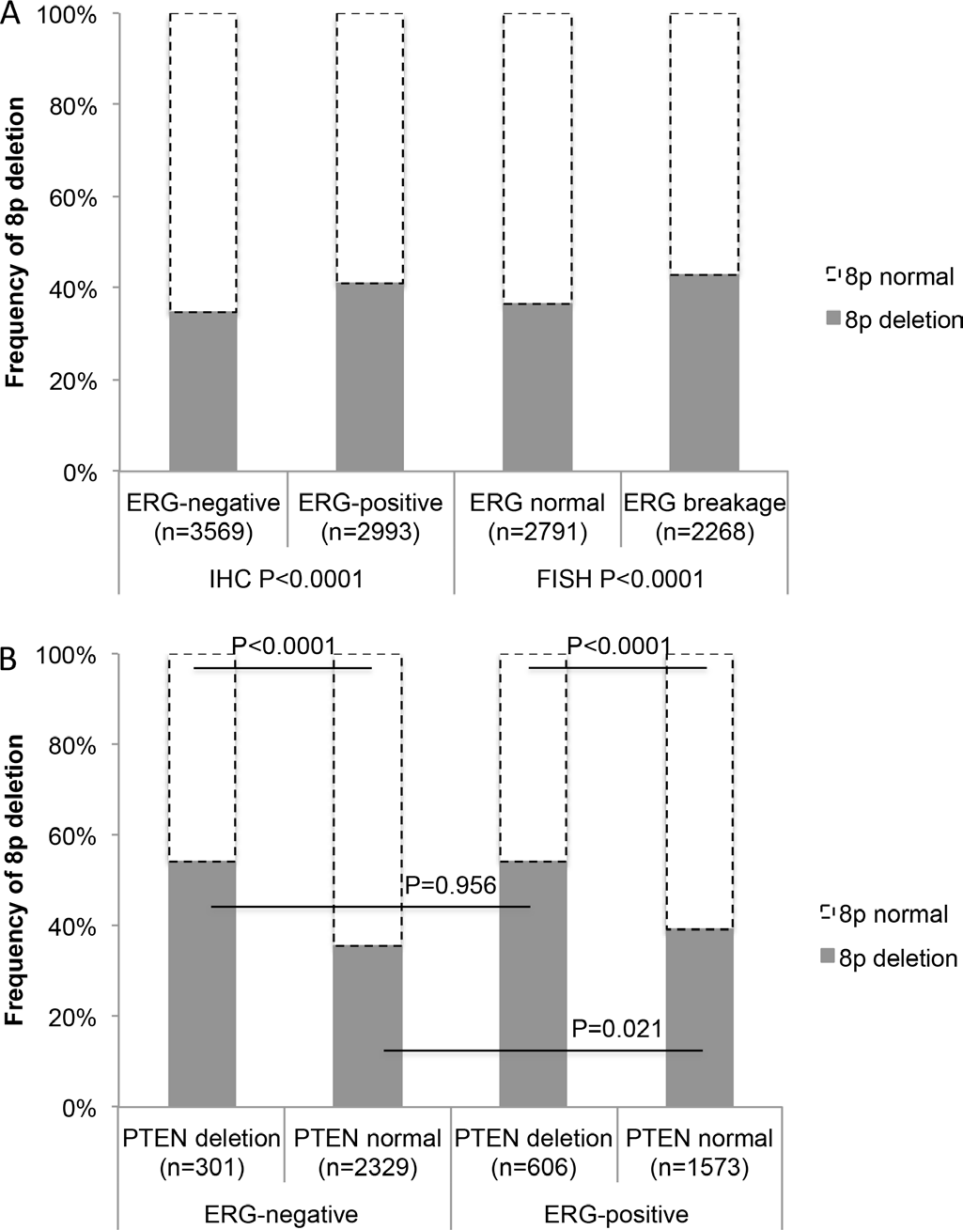
(**A**) Associations between 8p deletion and ERG-fusion by IHC and FISH analysis (**B**) Association between deletion of 8p and *PTEN* in ERG-negative and ERG-positive prostate cancers.

8p deletions were marginally more frequent in ERG-positive cancers (40.9% according to IHC and 42.9% to FISH) than in ERG-negative cancers (34.7% according to IHC and 36.4% to FISH%, Figure 2A). To better understand the impact of *PTEN* deletions on this association we compared the 8p deletion frequency in subsets of cancers defined by their *PTEN* deletion and *ERG* fusion status (Figure 2B). This analysis revealed that 8p deletions were massively linked to *PTEN* deletions independently of the ERG status (*P* < 0.0001 each in subsets of ERG-positive and ERG-negative cancers), while the ERG status had no relevant further impact on the 8p deletion frequency, neither in cancers with normal *PTEN* copy numbers (*P* = 0.021) nor in cancers with *PTEN* deletion (*P* = 0.956).

### Associations with prognosis

**Figure 3:**
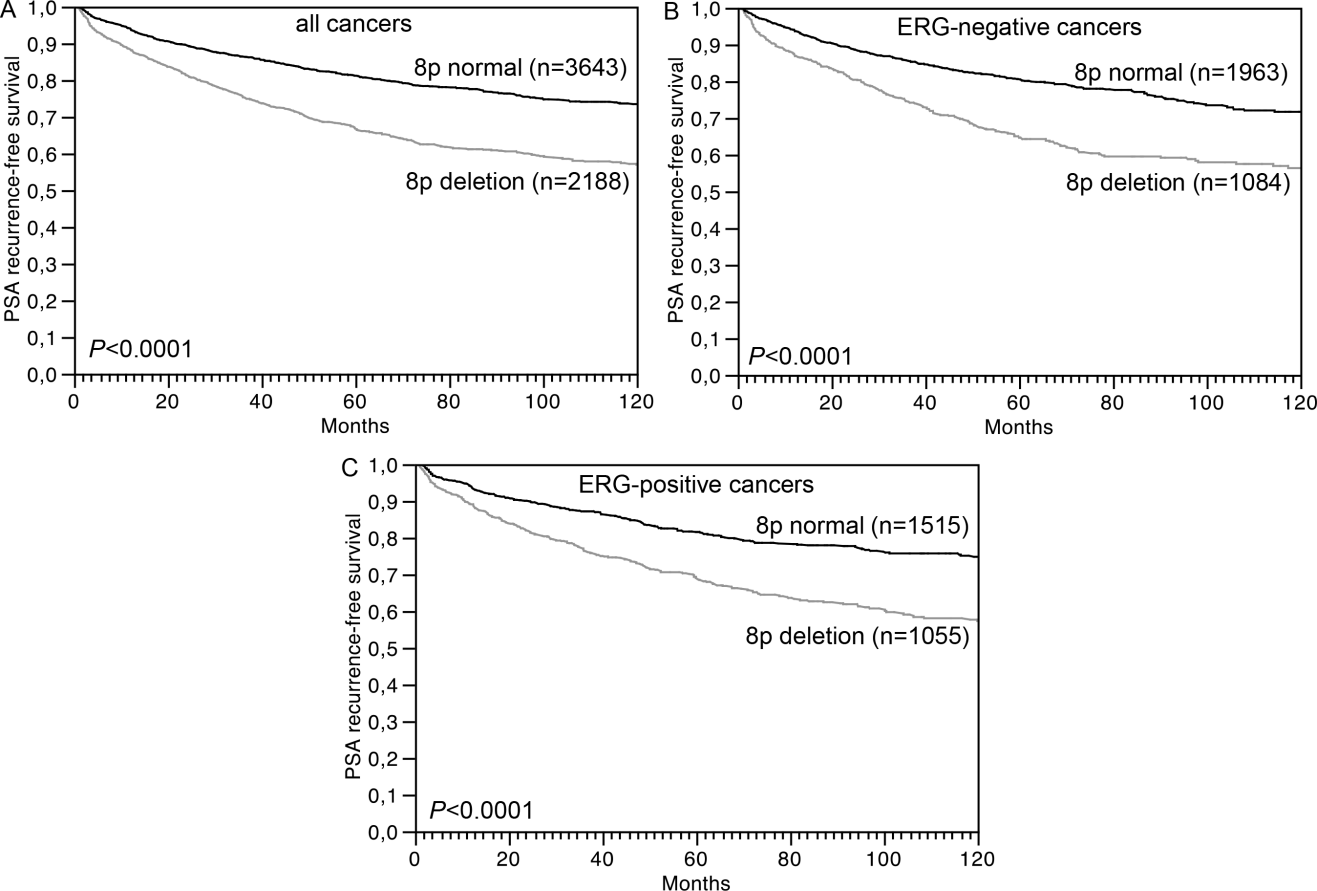
Association between 8p deletion and biochemical (PSA) recurrence in (A) all cancers (*n* = 6,375), (B) ERG-negative cancers (*n* = 3,231), and (C) ERG-positive cancers (*n* = 2,738)

The prognostic impact of pT and pN category as well as “classical” Gleason grade groups and quantitative Gleason grade is given for the 6,375 patients with interpretable 8p FISH for comparison (*P* < 0.0001 each, Supplementary Figure S1A–S1D). 8p deletion was significantly linked to early biochemical (PSA) recurrence in 6,375 cancers with interpretable 8p FISH results and follow-up data (*P* < 0.0001, Figure 3A). Multivariate analysis revealed that this was independent from established prognostic parameters including pathological tumor stage, Gleason grade, presence of lymph node metastases, status of the resection margin and pre-operative PSA level (*P* = 0.0100, Table 3). The relationship with patient outcome was similar in the subsets of 3,231 ERG-negative (*P* < 0.0001, Figure 3B) and 2,738 ERG-positive cancers (*P* < 0.0001, Figure 3C).

**Table 3:** Multivariate analysis (Cox regression) including clinical and pathological parameters in addition to the 8p deletion status in all prostate cancers

Parameter	RR	95% CI	*P*-value
**Tumor stage**			
pT3a vs pT2	2.0	1.7–2.3	< 0.0001
pT3b vs pT3a	1.4	1.2–1.7	
pT4 vs pT3b	2.1	1.4–2.8	
**Gleason grade**			
3 + 4 vs ≤ 3+ 3	2.3	1.8–3.0	< 0.0001
4 + 3 vs 3 + 4	2.1	1.8–2.4	
≥ 4 + 4 vs 4 + 3	1.2	1.0–1.5	
**Nodal stage**			
pN1 vs pN0	1.5	1.3–1.8	< 0.0001
**Resection margin status**			
R1 vs R0	1.2	1.0–1.3	0.0254
**Pre-operative PSA (ng/ml)**			
4–10 vs < 4	1.1	0.9–1.4	< 0.0001
10–20 vs 4–10	1.2	1.0–1.4	
> 20 vs 10–20	1.4	1.2–1.7	
**8p status**			
8p deletion vs 8p normal	1.2	1.0–1.3	0.0100

**Figure 4:**
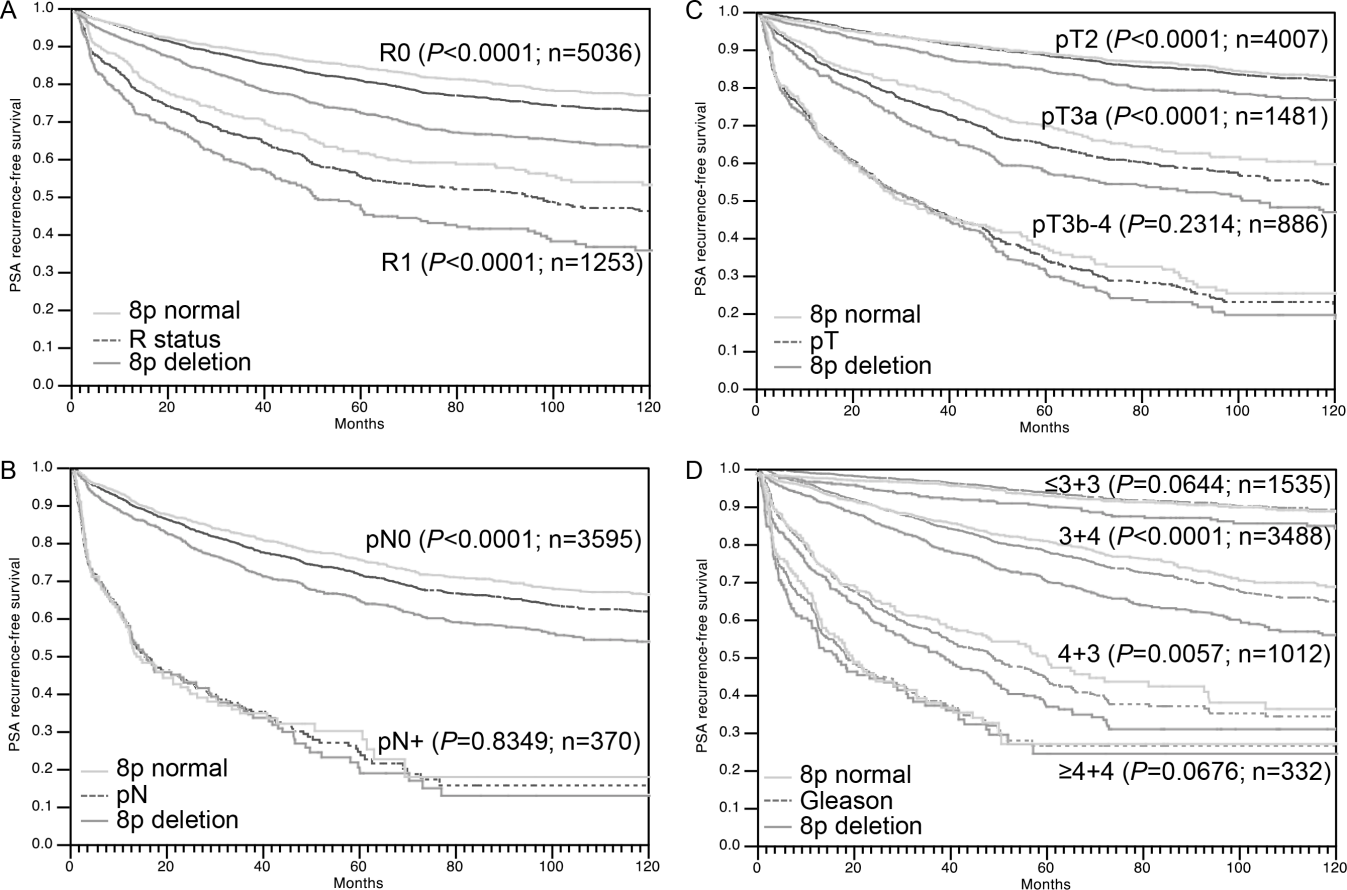
Association between 8p deletion and biochemical (PSA) recurrence in dependence on (A) resection margin status (R), (B) pathological lymph node status (pN), (C) pathological tumor stage(pT) and (D) classical Gleason grade

**Figure 5:**
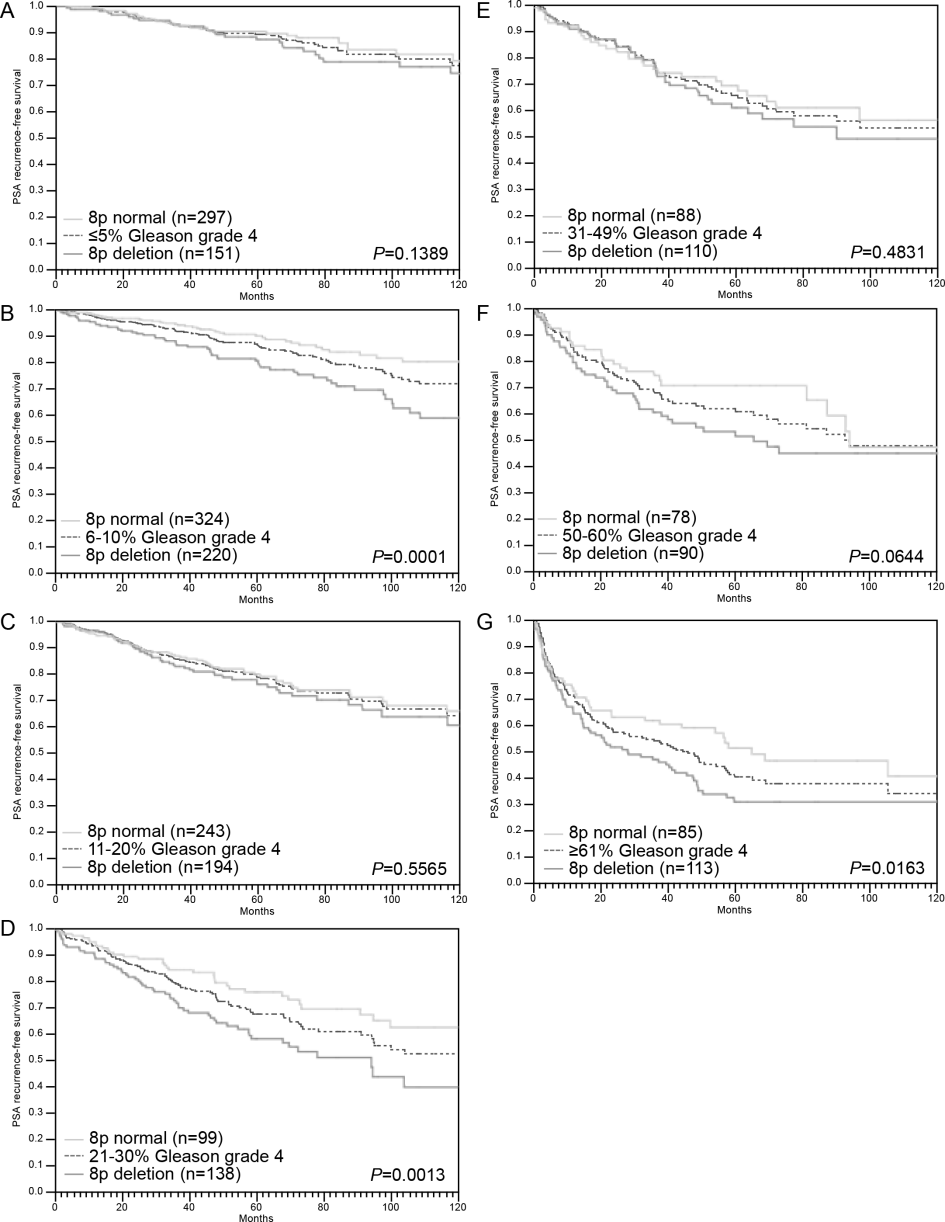
Association between 8p deletion and biochemical recurrence in dependence on quantitative Gleason grading subgroups (A–G)

A strong prognostic impact was also seen for the subgroups R0 and R1 cancers (Figure 4A). However, a variable impact on prognosis was seen for tumors with different pN stage (Figure 4B), pT stage (Figure 4C) or “classical” Gleason grade groups (Figure 4D). Here, 8p deletions could not distinguish prognostic subgroups in categories with a particular good (Gleason 3 + 3) or bad prognosis (Gleason ≥ 8, pN1, pT3b). A further analysis of tumors characterized by a comparable quantitative Gleason grade revealed, that the prognostic impact of 8p deletions was only retained in a few subgroups including cancers with 6–10%, 21–30% and ≥ 61% Gleason 4 (Figure 5A–5G).

**Figure 6:**
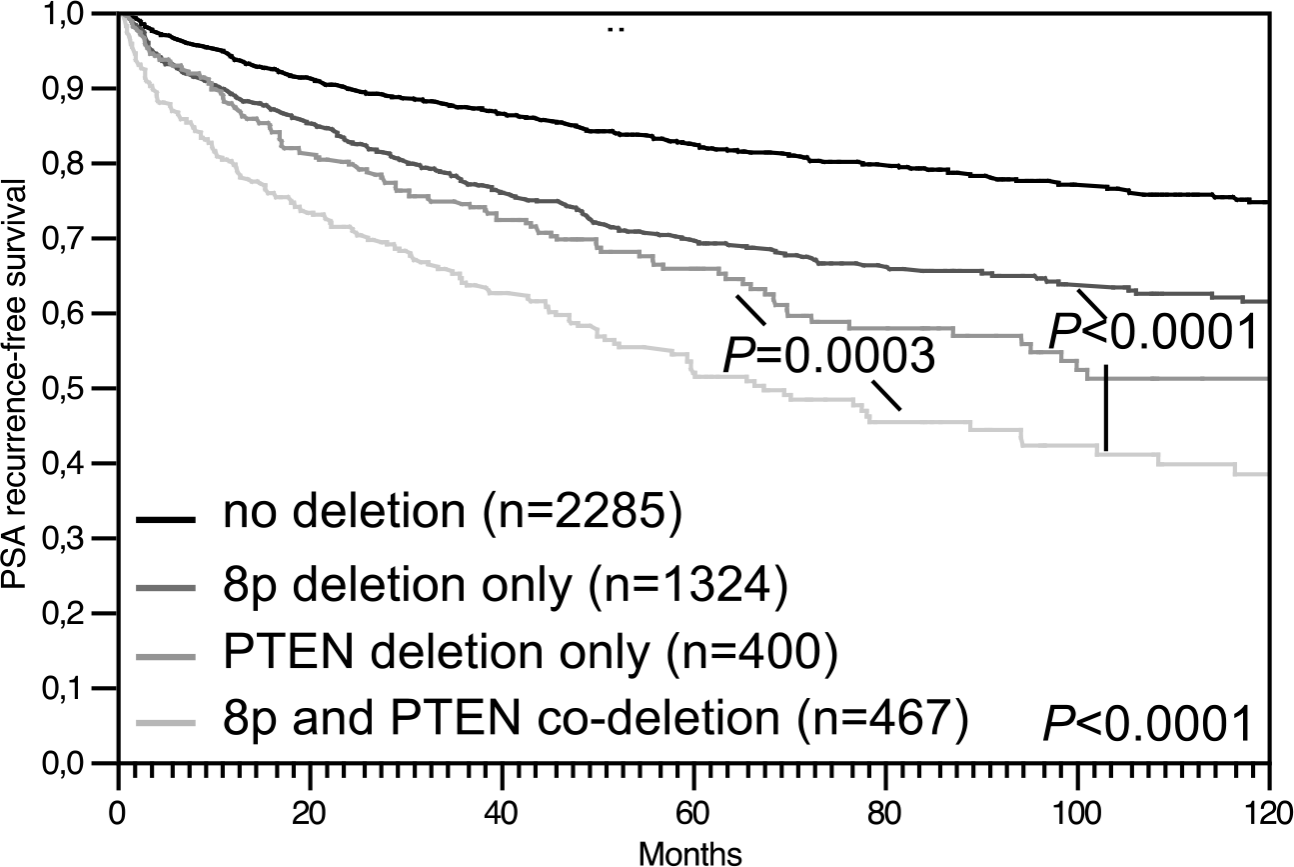
Association between 8p/PTEN co-deletion and biochemical (PSA) recurrence in all prostate cancers

Because of the strong association between 8p deletion and *PTEN* deletion, the prognostic impact of both deletions was also studied in combination (Figure 6). In this analysis, prognosis deteriorated continuously from 2,285 cancers without deletions to 1,324 cancers with 8p deletion only, to 400 cancers with *PTEN* deletion only, to 467 cancers with 8p/*PTEN* co-deletions (*P* = 0.0003 for *PTEN* deletion vs. 8p/*PTEN* co-deletion).

## DISCUSSION

Chromosome 8p deletion is one of the most frequent alterations in many different cancer types (reviewed in [20]). 8p deletions are typically large and often involve the entire short arm of the chromosome. This made the search for 8p deletion target genes difficult. Extensive studies searching for the underlying tumor suppressor gene have failed to identify a universal 8p tumor suppressor but have revealed multiple 8p genes with documented tumor suppressive properties, such as *NKX3.1* [21, 22], *CSMD1* [23], *DLC1* [24], *PPP2CB* and *PPP3CC* [25], *MSR1* [26], *TNFRSF10C* and *TNFRSF10D* [25]. Studies employing whole genome or exome sequencing have meanwhile clarified that 8p deletion is - in the vast majority of cases - not accompanied by a mutation of the coding area of any 8p gene [27–30]. It is still possible that one or several 8p-deleted genes are completely silenced by epigenetic or other mechanisms. It is an appealing alternative, however, that reduced expression of a combination of 8p genes could exert biologically relevant consequences in case of heterozygous 8p deletion. Studies in murine models of hepatocellular carcinomas, another cancer type frequently affected by large 8p deletions, have shown that partial inactivation of several 8p genes can cooperatively drive cancer development [31], thus supporting a model of compound haplo-insufficiency which might also apply for 8p-deleted prostate cancers. A significant biologic impact of haplo-insufficiency has recently also been demonstrated for *NKX3.1* [32].

Our meta-analysis of 8p copy number data demonstrates that deletions are typically large and often involve loss of the entire 8p chromosome arm. The successful analysis of more than 7,000 prostate cancers using a FISH probe directed against the *NKX3.1* locus, thus, indicates 8p deletions in 37% of tumors. This frequency is comparable to the 32% 8p deletions found in an own earlier study on a subset of 2,097 of our cancers [10] but somewhat lower than in most other FISH studies, which reported deletion frequencies between 40 and 69% [11–13, 33–35]. Substantially lower deletion rates (1.9%-15.6%) have been reported from studies on 56-333 prostate cancers analyzed within the frameworks of The Cancer Genome Atlas (TCGA) and the International Cancer Genome Consortium (ICGC) [36, 37]. These discrepant findings are most likely attributable to the comparatively small sample sets and the different thresholds for 8p deletion calling. For example, reported deletion rates in the TCGC/ICGC data portal are limited to “deep deletions” extending a certain threshold (−2) in Genomic Identification of Significant Targets in Cancer (GISTIC) analysis [36, 37], while “shallow deletions” (threshold −1) are not reported. Although “deep” and “shallow” deletions are defined as “possibly homozygous” and “possibly heterozygous” deletions (http://www.cbioportal.org [36, 37]) such data are difficult to compare with FISH findings that allow for precise copy number counting. The higher deletion rates in earlier FISH studies are likely due to the use of thresholds for defining 8p deletions that were based on FISH results in normal epithelial cells [11, 13, 33]. This is a potential source of error, because cancer cell nuclei are markedly larger than nuclei from normal epithelial cells and consequently, the rate of FISH signal loss through nuclear truncation will generally be higher in cancer cells than in normal epithelial cells on tissue sections. The cut-off levels selected for our FISH deletion analyses are based on a 100% concordance with CGH array data found for *PTEN* deletions in an earlier study [38]. High rates of 8p deletion were also described in studies using conventional or array-based comparative genomic hybridization (CGH). For example, two meta-analysis of 872 and 622 prostate cancers analyzed by classical CGH [5] or array CGH (aCGH) [6] revealed 34%-62% 8p deletions, and a large array CGH study reported 53–78% 8p deletions in 181 prostate cancers [4]. In a comparison with studies from our own laboratory using identical criteria for FISH deletion scoring, 8p deletion (36%) is the second most frequent genomic alteration right after *TMPRSS2*:*ERG* fusions (52%). The next frequent deletions are 16q (21%) [39], *PTEN* (20%) [38] and 6q (18%) [40].

Our data identify 8p deletions as a strong prognostic parameter, which is independent of established clinical and pathological prognosticators. This is generally in line with earlier studies on 27-2,097 patients suggesting associations of 8p deletions with unfavorable tumor phenotype [5–12], metastasis [8, 12], hormone refractory disease [10], and PSA recurrence [10, 13]. The high number of cancers analyzed in this study enabled a selective analysis of clinically relevant subgroups. Here, the complete absence of a prognostic impact of 8p deletions in certain high-risk subpopulations such as pT3b, N1 or Gleason ≥ 8 cancers demonstrates, that the dismal prognosis of these cancers is already quite reliably recognized by established histological parameters. That 8p deletion does not distinguish a particularly aggressive subgroup among these tumors further suggests, that 8p deletion does not lead to the activation of a “key prognostic pathway”, which – even in advanced cancers - would result in a massive tumor growth or devastating metastatic spread.

The variable prognostic impact of 8p deletions in cancers with different Gleason grades is of particular interest. That 8p deletions were prognostically most relevant in the subgroup of 3 + 4 carcinomas in univariate as well as in multivariate analysis (when combined with Gleason score) is encouraging because Gleason 3 + 4 represents the clinically most difficult group with treatment options ranging from active surveillance to prostatectomy. These data also illustrate, that the subgroup Gleason 3 + 4 is more heterogeneous then others in clinical outcome. Based on the morphologic analysis of more than 10.000 prostate cancers, we had recently shown, that prognostic Gleason Grade information can be expanded in Gleason 3 + 4 and 4 + 3 cancers by using the percentage of Gleason 4 patterns as a continuous variable. Both in biopsies and in prostatectomy samples, prostate cancer prognosis continuously deteriorated with increasing percentage of Gleason 4 pattern (quantitative Gleason Grade) [41]. That a statistically significant prognostic impact of 8p deletion only remained in few subgroups defined by a comparable quantitative Gleason grade illustrates how difficult it is for a molecular parameter to outperform morphological parameters of malignancy in prostate cancer.

That 8p deletions were not pinpointing towards an unfavorable clinical course in Gleason 3 + 3 carcinomas is of particular interest. It demonstrates that a morphologically benign appearing prostate cancer is not necessarily aggressive, if molecular features with documented prognostic relevance are detected. The high frequency of 8p deletion in 3 + 3 carcinomas (25%) further argues against a clinical importance of this alteration in such early cancers. The fraction of 3 + 3 carcinomas that are thought to develop an aggressive disease course is far lower than 25% [42]. The strong (and independent) overall prognostic impact of 8p deletions in prostate cancer in combination with the high frequency but lack of clinical relevance in 3 + 3 cancers leads us to hypothesize, that 8p deletion represents an early alteration in prostate cancer needing additional factors to drive a cancer towards high aggressiveness.

Our data suggest *PTEN* inactivation as a possible candidate for a molecular aberration that could particularly efficiently interplay with 8p deletion. *PTEN* deletion is the strongest single molecular prognostic feature in prostate cancer known as to yet. The strong statistical association between *PTEN* and 8p deletions and the particularly poor clinical course in case of a co-deletion of *PTEN* and 8p argues for synergistic effects between these lesions. The 8p genes possibly involved in such an interaction remain unclear. However, mouse models suggest that *PTEN* loss could drive invasion and metastasis in cooperation with loss of *NKX3.1* [43, 44]. Earlier studies had demonstrated that the panel of chromosomal deletions occurring in prostate cancer is largely dependent on the ERG status. Deletions of *PTEN*, 16q, and 3p13 are tightly linked to ERG expression while deletions of 6q15 and 5q21 is largely restricted to ERG-negative cancers [4, 7, 15, 27, 38, 40, 45, 46]. The data of the present study delineate 8p deletion as the exceptional case of an “ERG-independent” deletion. This is also supported by CGH array data from a cohort of 181 prostate cancers, where no significant link between 8p deletion and *ERG* fusion was reported [4]. Our data demonstrate, that the slightly higher rate of 8p deletions in ERG-positive (40.9%) than in ERG-negative cancers (34.7%) is entirely driven by its strong association with the ERG-linked *PTEN* deletion.

In summary, 8p deletion is the second most common genomic alteration in prostate cancer. Several lines of evidence suggest an interplay with *PTEN* deletions. 8p deletion is a strong and independent prognostic factor in prostate cancer. That 8p deletion lacks prognostic significance in several morphologically defined subgroups with very poor or very good prognosis demonstrates the power of established criteria – such as the Gleason grade - for assessing prostate cancer aggressiveness.

## MATERIALS AND METHODS

### Patients

A set of prostate cancer tissue microarrays (TMA) was used in this study containing one tissue core each from 12,427 consecutive radical prostatectomy specimens from patients undergoing surgery at the Department of Urology, and the Martini Clinic, Prostate Cancer Center, University Medical Center Hamburg-Eppendorf. This TMA is based on our previous 3,261 samples prostate prognosis TMA [10], with additional 9,166 tumors and updated clinical data from 12,344 patients with a median follow-up of 36.4 months (range: 1 to 241 months; Table 4). In all patients, prostate specific antigen (PSA) values were measured quarterly in the first year, followed by biannual measurements in the second and annual measurements after the third year following surgery. Recurrence was defined as a postoperative PSA of 0.2 ng/ml and rising thereafter. The first PSA value above or equal to 0.2 ng/ml was used to define the time of recurrence. Patients without evidence of tumor recurrence were censored at the time of the last follow-up. All prostate specimens were diagnosed according to a standard procedure, including complete embedding of the entire prostate for histological analysis [47]. The TMA manufacturing process was described earlier in detail [48, 49]. In short, one 0.6 mm core was taken from a representative tissue block from each patient. The tissues were distributed among 27 TMA blocks, each containing 144 to 522 tumor samples. Presence or absence of cancer tissue was validated by immunohistochemical AMACR and 34BE12 analysis on adjacent TMA sections. For internal controls, each TMA block also contained various control tissues, including normal prostate tissue. The molecular database attached to this TMA contained results on ERG expression in 10,678, *ERG* break apart fluorescence *in-situ* hybridization (FISH) analysis in 7,099 (expanded from [27, 50]), and deletion status of *PTEN* in 6,704 (expanded from [38]) tumors.

**Table 4:** Clinico-pathological features of 12,427 arrayed prostate cancers

	No. of patients (%)	
	Study cohort on TMA (*n* = 12427)	Biochemical relapse among categories
**Follow-up (mo)**		
*n*	11665 (93.9%)	2769 (23.7%)
Mean	48.9	-
Median	36.4	-
**Age (y)**		
≤ 50	334 (2.7%)	81 (24.3%)
51–59	3061 (24.8%)	705 (23.0%)
60–69	7188 (58.2%)	1610 (22.4%)
≥ 70	1761 (14.3%)	370 (21.0%)
**Pre-operative PSA (ng/ml)**		
< 4	1585 (12.9%)	242 (15.3%)
4–10	7480 (60.9%)	1355 (18.1%)
10–20	2412 (19.6%)	737 (30.6%)
> 20	812 (6.6%)	397 (48.9%)
**pT category (AJCC 2002)**		
pT2	8187 (66.2%)	1095 (13.4%)
pT3a	2660 (21.5%)	817 (30.7%)
pT3b	1465 (11.8%)	796 (54.3%)
pT4	63 (0.5%)	51 (81.0%)
**Gleason grade (WHO/ISUP 2016)**		
< 7	2997 (24.2%)	368 (12.3%)
3 + 4	6964 (56.1%)	1288 (18.5%)
4 + 3	1849 (14.9%)	789 (42.7%)
8	127 (1.0%)	50 (39.4%)
9–10	469 (3.8%)	262 (55.7%)
**pN category**		
pN0	6970 (91.0%)	1636 (23.5%)
pN+	693 (9.0%)	393 (56.7%)
**Resection margin status**		
Negative	9990 (81.9%)	1848 (18.5%)
Positive	2211 (18.1%)	853 (38.6%)

NOTE: Numbers do not always add up to 12427 in the different categories because of cases with missing data. Abbreviation: AJCC, American Joint Committee on Cancer.

The usage of archived diagnostic left-over tissues for manufacturing of tissue microarrays and their analysis for research purposes as well as patient data analysis has been approved by local laws (HmbKHG, §12,1) and by the local ethics committee (Ethics commission Hamburg, WF-049/09 and PV3652). All work has been carried out in compliance with the Helsinki Declaration.

### Fluorescence *in-situ* hybridization

**Figure 7:**
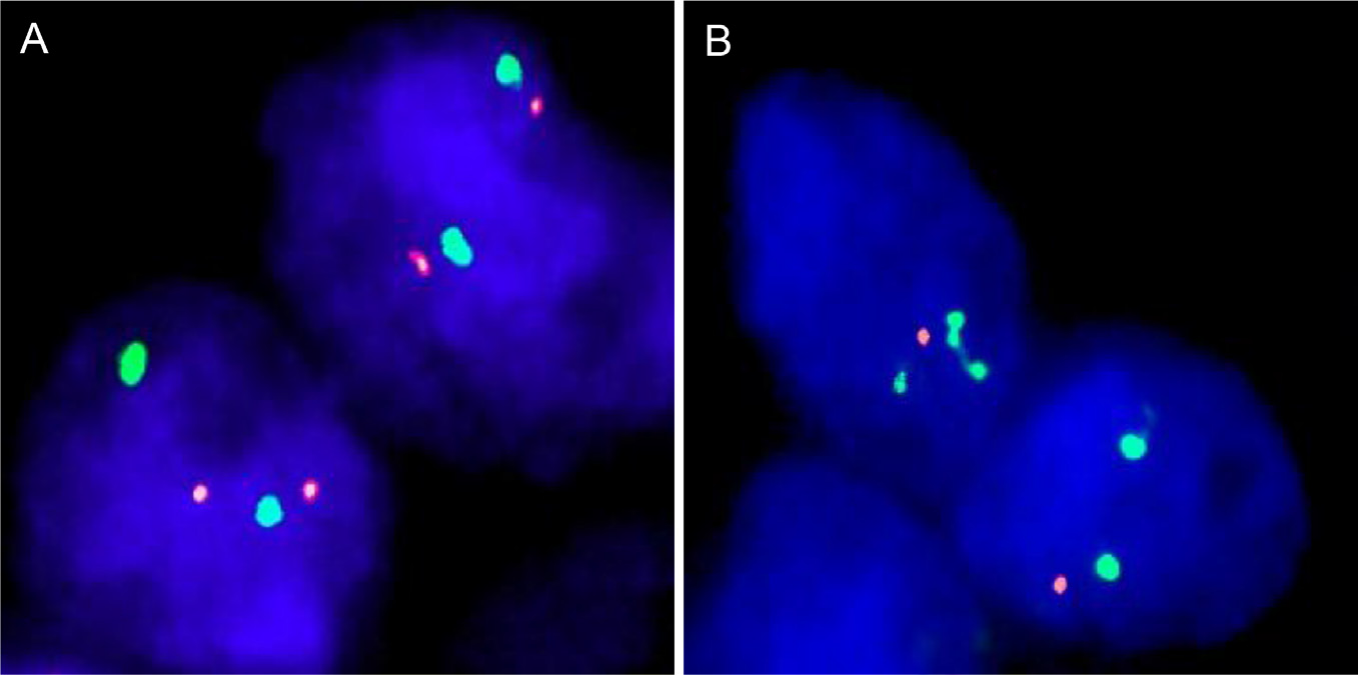
Examples of FISH findings using the 8p deletion probe (**A**) Normal 8p copy numbers as indicated by two orange 8p signals and two green centromere 8 signals. (**B**) Heterozygous deletion as indicated by the lack of one orange 8p signal.

Four micrometer TMA sections were used for fluorescence *in-situ* hybridization (FISH). For proteolytic slide pretreatment, a commercial kit was used (paraffin pretreatment reagent kit; Abbott, Chicago, USA) TMA sections were deparaffinized, air-dried, and dehydrated in 70%, 85%, and 100% ethanol, followed by denaturation for 5 min at 74°C in 70% formamid 2× SSC solution. The FISH probe set consisted of a spectrum-orange labeled *NKX3.1* probe (made from a mixture of BAC RP11-625E02 and BAC RP11-116M17), and a commercial spectrum-green labeled centromere 8 probe (#6J37-08; Abbott, Chicago, USA) as a reference. Hybridization was overnight at 37°C in a humidified chamber. Slides were subsequently washed and counterstained with 0.2μmol/L 4′-6-diamidino-2-phenylindole in antifade solution. Stained slides were manually interpreted with an epifluorescence microscope, and the predominant FISH signal numbers were recorded in each tissue spot. Homozygous deletion of 8p was defined as complete absence of *NKX3.1* FISH probe signals in ≥ 60% of tumor nuclei, with the presence of one or two *NKX3.1* FISH signals in adjacent normal cells. Tissue spots with a lack of *NKX3.1* signals in all (tumor and normal cells) or lack of any normal cells as an internal control for successful hybridization of the *NKX3.1* probe were excluded from analysis. Heterozygous deletion of *NKX3.1* was defined as the presence of fewer *NKX3.1* signals than centromere 8 probe signals of ≥ 60% tumor nuclei (Figure 7). These thresholds were based on a previous study comparing *PTEN* deletion data obtained by FISH and SNP in a subset of cancers included into this TMA set [38].

### 8p copy number data sources and analysis

Raw data were obtained from 4 large studies employing array CGH or SNP array analysis in a total of 442 prostate cancers [4, 15–17]. Data were imported into the FISH Oracle browser [18, 19] and visualized in different tracks corresponding to each study. A global threshold of −0.3 was applied to all 4 datasets to display deletions.

### Statistics

For statistical analysis, the JMP 9.0 software (SAS Institute Inc., NC, USA) was used. Contingency tables and Chi-square (Likelihood) tests were utilized to study the relationship between 8p deletion and categorical clinico-pathological variables. Kaplan Meier curves were generated for PSA recurrence free survival. The log-Rank test was applied to test the significance of differences between stratified survival functions. Cox proportional hazards regression analysis was performed to test the statistical independence and significance between pathological, molecular, and clinical variables. Because of the high number of samples included in our study, we considered differences as being statistically relevant at an alpha niveau of 0.01.

## SUPPLEMENTARY MATERIALS


