# Supplementary material for: Deletion of 8p is an independent prognostic parameter in prostate cancer
Source: Oncotarget. 2016 Nov 17;8(1):379–92. doi: 10.18632/oncotarget.13425 (PMC5352127; doi:10.18632/oncotarget.13425)
Supplement: Supplementary file 1 [file oncotarget-08-379-s001.pdf]

Deletion of 8p is an independent prognostic parameter in prostate cancer

Supplementary Materials

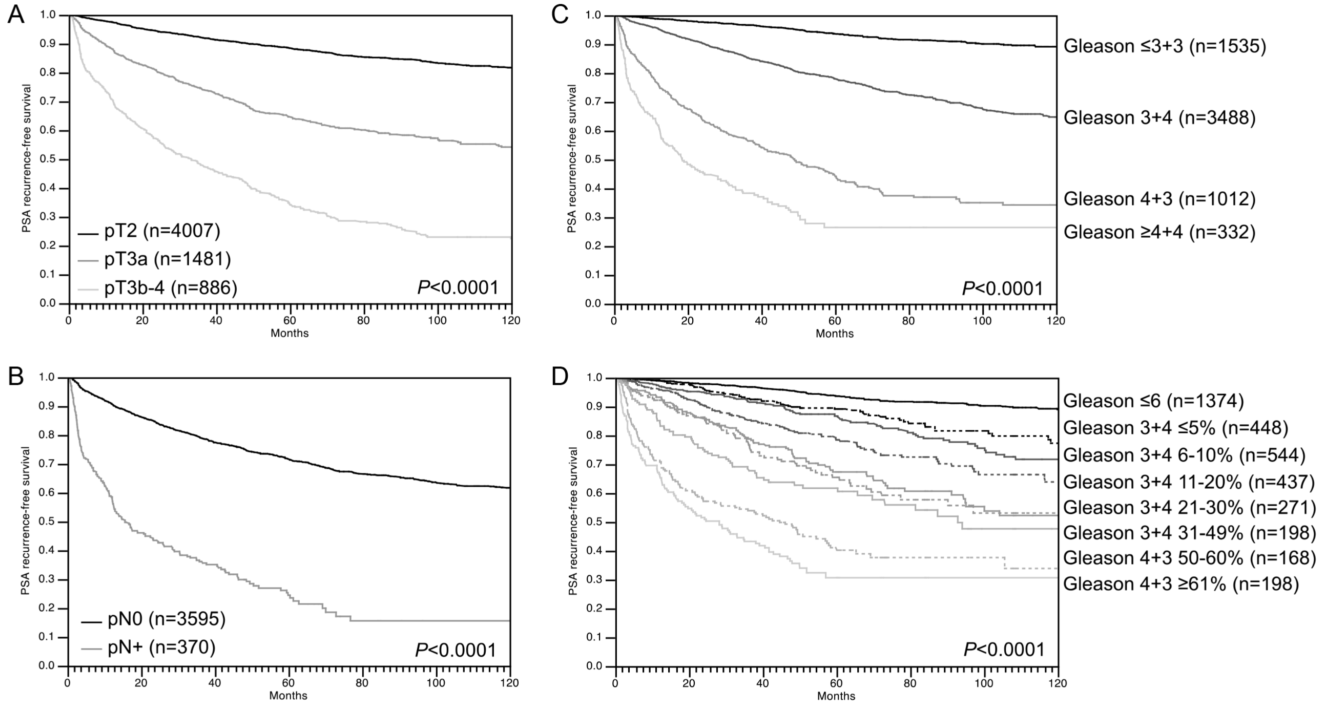

Supplementary Figure S1: Prognostic impact of (A) pathological tumor stage (pT), (B) pathological lymph node status (pN), (C) classical Gleason grade groups (WHO/ISUP 2016) and (D) quantitative Gleason grade in the subset of 6,375 prostate cancers with interpretable 8p FISH results.
